# IL-6-stimulated CD11b^+^CD14^+^HLA-DR^−^ myeloid-derived suppressor cells, are associated with progression and poor prognosis in squamous cell carcinoma of the esophagus

**DOI:** 10.18632/oncotarget.2368

**Published:** 2014-08-19

**Authors:** Miao-Fen Chen, Feng-Che Kuan, Tzu-Chen Yen, Ming-Shian Lu, Paul-Yang Lin, Yi-Hsiu Chung, Wen-Cheng Chen, Kuan-Der Lee

**Affiliations:** ^1^ Department of Radiation Oncology, Chang Gung Memorial Hospital at Chiayi, Taiwan; ^2^ Chang Gung University, College of Medicine, Taiwan; ^3^ Hematology and Oncology, Chang Gung Memorial Hospital at Chiayi, Taiwan; ^4^ Nuclear Medicine Department, Chang Gung Memorial Hospital at Linko, Taiwan; ^5^ Center for Advanced Molecular Imaging and Translation, Chang Gung Memorial Hospital, at Linko, Taiwan; ^6^ Thoracic & Cardiovascular Surgery, Chang Gung Memorial Hospital at Chiayi, Taiwan; ^7^ Pathology, Chang Gung Memorial Hospital at Chiayi, Taiwan

**Keywords:** IL-6, esophageal SCC, MDSC

## Abstract

The aim of this study was to assess the significance of myeloid-derived suppressor cells (MDSCs) and their association with IL-6 in esophageal squamous cell carcinoma (SCC). We examined the percentage of CD11b+CD14^+^HLA-DR^−^ myeloid cells and the levels of IL-6 in the peripheral blood of 50 patients with esophageal SCC and 12 healthy controls. Moreover, we evaluated the relationship between MDSC recruitment, IL-6 levels, and tumor progression by adding 4-nitroquinoline 1-oxide (4-NQO) to the drinking water of mice to induce esophageal tumors. Here we demonstrated that circulating CD11b+CD14^+^HLA-DR^−^ cells were significantly increased in esophageal SCC patients compared with healthy people, and this was associated with the clinical stage, treatment response and circulating IL-6 levels. In a 4-NQO-induced esophageal tumor animal model, MDSC recruitment was associated with invasive esophageal tumors and with increased IL-6 levels. IL-6 stimulated reactive oxygen species, arginase 1 and p-STAT3 in MDSCs. Blockade of IL-6 prevented induction of MDSCs and the incidence of 4-NQO- induced invasive tumors. In conclusion, the levels of MDSCs and IL-6 predicted the prognosis of patients with esophageal SCC. Moreover, we suggest inhibition of IL-6 as a potential strategy for the treatment of esophageal SCC.

## INTRODUCTION

Esophageal cancer is an aggressive upper gastrointestinal malignancy that generally present as a locally advanced tumor that requires multimodal treatment [1]. Identification of the potential molecular markers for predicting the treatment response and understanding the molecular mechanisms underlying aggressive tumor growth is important for the effective management and prognosis of esophageal cancer.

Abundant epidemiological data revealed a strong correlation between inflammation and the incidence of cancers [2, 3]. Chronic inflammation contributes to tumor initiation and progression via both non-immune and immune mechanisms [4]. Most cancers are characterized by the overproduction of immunosuppressive cells and cytokines [5]. Myeloid-derived suppressor cells (MDSCs) are an immature population of myeloid cells that are thought to be an important subset of cells that contribute to an immunosuppressive tumor microenvironment [6-8], and significantly increased in number in cancer patients. A significant correlation was reported among circulating MDSCs, metastatic burden, and clinical stage in some tumors, including gastrointestinal cancers [9-11]. There are two distinct histological types of esophageal cancer: squamous cell carcinoma (SCC), and adenocarcinoma. These subtypes are significantly different in terms of their incidence, natural history, and treatment outcomes [12]. Different human tumors induce different subtypes of MDSCs. Although abnormal accumulation of MDSC subsets was reported to be elevated in patients with GI malignancy, it remains unclear how MDSCs are regulated and how they modulate the biological activities and prognosis of esophageal SCC.

In recent studies, a novel subset of MDSCs were identified based on the presence of CD14 expression but the absence of human leukocyte antigen (HLA)-DR expression (CD14^+^HLA-DR^−^) in the peripheral blood of cancer patients, including those with lung cancer and head and neck SCC [13-15]. The abnormal accumulation of CD14^+^HLA-DR^−^ cells reportedly contributes to tumor immune evasion and correlates with cancer prognosis. Therefore, we focused our work to assess the predictive value of CD11b+CD14^+^HLA-DR^−^ myeloid cells in patients with esophageal SCC. We also investigated the potential role of MDSCs in tumor progression using a 4-nitroquinoline 1-oxide (4-NQO)-induced esophageal tumor animal model, which could study the effects of immunity on esophageal cancer in immunocompetetnt mice [16]. In addition, MDSCs are induced and/or activated by proinflammatory mediators [17-19]. We reported previously that IL-6 correlated significantly with poor prognosis in esophageal SCC patients [20]. If inflammation-induced MDSCs are an important link between inflammation and cancer, then the downregulation of inflammatory factors could reduce MDSC levels and delay tumor progression. To assess this hypothesis, we evaluated the relationship between IL-6 and circulating MDSC subsets in patients, and the ability of IL-6 to stimulate MDSC accumulation in 4-NQO-induced esophageal tumor model to provide new insights into the development of immune-based therapy.

## RESULTS

### Peripheral CD11b^+^CD14^+^HLA-DR^-^ cells in patients with esophageal SCC

**Figure 1:**
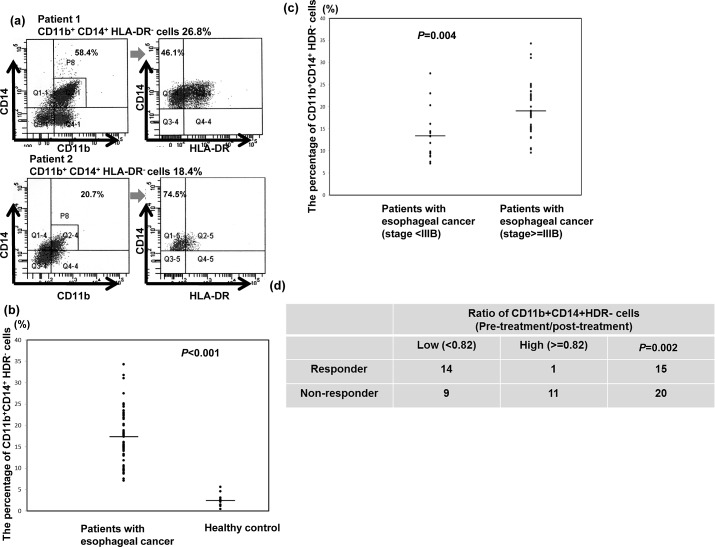
CD11bCD14HLA-DR cells in patients with esophageal SCC a. Flow cytometric analysis of circulating CD11b^+^CD14^+^HLA-DR^−^ cells in isolated PBMCs. CD11b+CD14+ cells were gated, and the HLA-DR negative population was then selected. Representative data from two cancer patients are shown. b. Frequency of circulating CD11b^+^CD14^+^HLA-DR^−^ cells in PBMCs isolated from cancer patients (n=50) and healthy donors (n=12). The lines indicate the mean values. c. Frequency of circulating CD11b^+^CD14^+^HLA-DR^−^ cells in PBMCs isolated from cancer patients with stage II and IIIa (n=15) and stage IIIb and IV (n=35) disease. Lines indicate the mean values. d. Differences in the treatment response according to the mean percentage of CD11b^+^CD14^+^HLA-DR^−^ cells after treatment relative to that at diagnosis (82%) in the high (ratio after treatment ≥ 82%) and low groups (< 82%). (The ratio means the percentage of CD14+HLA-DR− cells at post-treatment/ the percentage of CD14+HLA-DR− cells at diagnosis)

The percentage of CD11b^+^CD14^+^HLA-DR^−^ cells in the peripheral circulation of patients with esophageal SCC and healthy controls was evaluated using flow cytometry. Blood from the 50 patients was obtained within 1 month of cancer diagnosis, and none of the individuals had received prior cancer therapy. PBMCs were isolated from esophageal cancer patients or healthy donors and were stained for detecting MDSCs using fluorochrome-labelled antibodies targeting CD14, CD11b, and HLA-DR. Representative flow cytometry data from two esophageal SCC patients are shown in Figure 1a. The mean percentage of CD11b^+^CD14^+^HLA-DR^−^ cells relative to the total number of CD11b+ myeloid cells in the PBMCs of esophageal SCC patients was 42.2±9.7% compared to 5.18±1.3% in healthy donors. Moreover, the percentage of CD11b^+^CD14^+^HLA-DR^−^ cells was significantly elevated in the cancer patients (17.3±6.4%) relative to the healthy donors (2.4±1.48%) (Fig. 1b). To evaluate the clinical significance of the CD11b^+^CD14^+^HLA-DR^−^ subset in the esophageal SCC patients, we further analyzed these cells according to clinical tumor parameters. The percentage of CD11b^+^CD14^+^HLA-DR^−^ cells among the PBMCs of patients with stage IIIb or IV tumors was higher than that of patients with stage II or IIIa disease (Fig. 1c). Moreover, to assess the usefulness of CD11b^+^CD14^+^HLA-DR^−^ cells for predicting treatment efficacy, 35 patients who completed planned treatment were divided into two groups according to mean CD11b^+^CD14^+^HLA-DR^−^ cell percentage after treatment relative to the level at diagnosis (82%): high (ratio after treatment ≥ 82%) versus low groups (< 82%). As shown in Figure 1d, 14 of the 23 patients in the low group were responders, while only one patient in the high group responded to treatment.

### MDSC levels correlate with tumor growth *in vivo*

**Figure 2:**
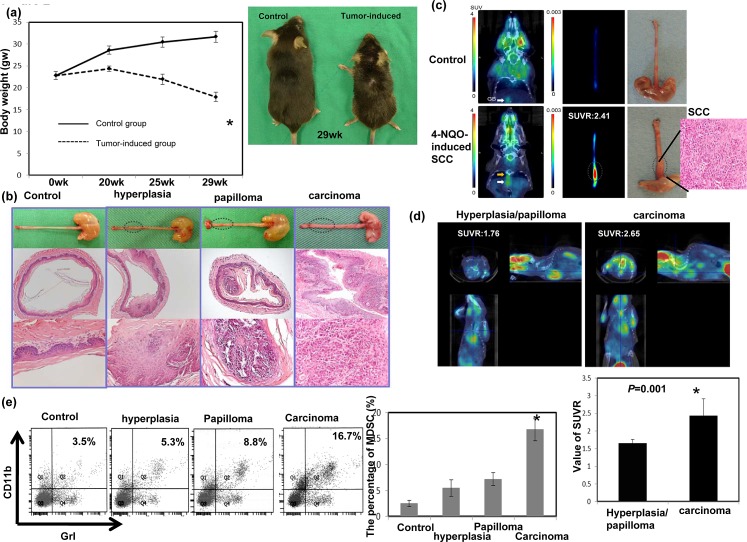
Evaluation of esophageal tumor formation using histological examination and micro animal PET imaging and its relationship with MDSC recruitment a. The body weights of animals that consumed drinking water containing the carcinogen 4-NQO or vehicle (propylene glycol, control). The data represent the means ± SEM of five animals per group at the indicated times. *, p<0.05. A representative image of mice 13 weeks after a 16-week 4-NQO treatment period is shown. b. Images of the gross lesions and pathological findings of tissue sections from the animals treated with 4-NQO or vehicle for 16 weeks that were followed for 12–14 weeks. Histological determinations included hyperplasia, papilloma, and invasive carcinoma. c. Representative PET images from the mice treated with 4-NQO and diagnosed with invasive carcinoma, compared with the control. The tumor-to-muscle SUVR was calculated from the micro-PET scans. d. Representative PET images from mice treated with 4-NQO and diagnosed with hyperplasia/papilloma or invasive carcinoma. The tumor-to-muscle SUVR was calculated from the micro-PET scans. Data points represent the means ± SEMs. *, p<0.05. e.Flow cytometric analysis of CD11b+Gr1+ cells from mice exhibiting hyperplasia/papilloma or invasive carcinoma after histological analysis. Representative images and quantitation are shown. The columns represent the means ± SEM. *, p<0.05.

Our clinical data suggested that the level of CD11b^+^CD14^+^HLA-DR^−^ myeloid cells was linked to the progression of esophageal SCC. Therefore, we examined the relationship between MDSCs and tumor progression using a 4-NQO-induced esophageal tumor mouse model. Esophageal SCC could be induced in mice by the consumption of drinking water containing 100 μg/ml 4-NQO for 16 weeks. Figure 2a shows that the animals that consumed 4-NQO-containing water lost weight progressively compared with those that drank water containing propylene glycol alone. At 12-14 weeks after the 16-week 4-NQO treatment and after the micro-PET images were taken, the mice were analyzed and their esophaguses removed for further evaluation. As shown in Figure 2b, tumor lesions on the esophagus were noted by gross examination and pathological analysis of the tissue sections. Pathological evidence of esophageal lesions, including hyperplasia, papilloma, and invasive carcinoma, was found in the 4-NQO-treated mice. The validity of micro-PET for detecting esophageal tumors in mice is shown in Figure 2c. The SUVR was calculated to represent the tumor lesion. Moreover, Figure 2d demonstrated that esophageal tumors with invasive carcinoma had a significantly higher SUVR than those with hyperplasia or papilloma only. Next, we assessed the link between tumor progression and MDSC recruitment in mice. The percentage of MDSCs was correlated significantly with invasive carcinoma formation in the treated mice (Fig. 2e).

### Effect of MDSC on tumor progression *in vivo*

**Figure 3:**
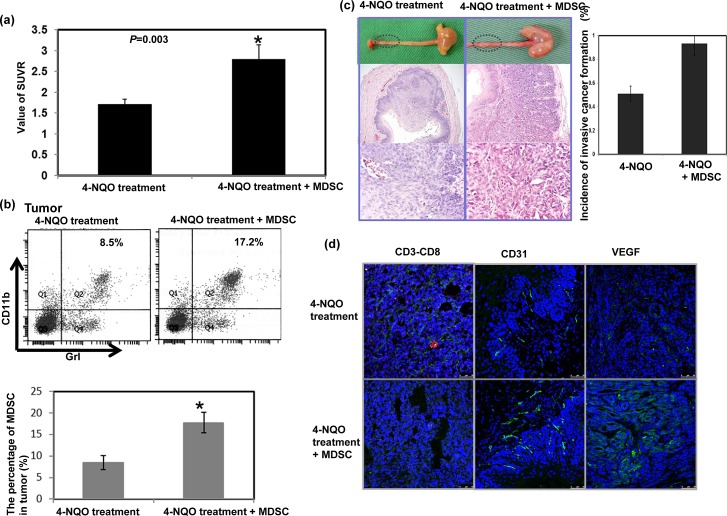
Effect of MDSCs on tumor progression *in vivo* a. The infusion of MDSCs increased the SUVR of PET images in esophageal lesions of mice followed for 12–14 weeks after a 16-week 4-NQO treatment period. The tumor-to-muscle SUVR was calculated from the micro-PET scans. Data points represent the means ± SEMs. *, p<0.05 b. Flow cytometric analysis of CD11b+Gr1+ cells from the 4-NQO-treated mice with or without MDSC infusion. Representative images and quantitative data are shown. The columns represent the means ± SEM. *, p<0.05. c. Representative images of gross lesions and the pathological findings on sectioned tissue samples from the 4-NQO-treated mice with or without MDSC infusion. Quantitative data assessing the incidence of invasive esophageal tumors are shown. The columns represent the means ± SEM. *, p<0.05 d. MDSC infusion attenuated the accumulation of CD3+CD8+ cells (Green: CD3; Red: CD8) and increased CD31 and VEGF immunostaining. Representative images are shown.

The role of MDSC accumulation in tumor progression was further examined in the 4-NQO-induced esophageal tumor with or without infusing sorted MDSCs. As shown in Figure 3a, MDSC infusion increased the SUVR in esophageal lesions in the mice by 12–14 weeks following the 16-week 4-NQO treatment. Histological examination and FACS analysis of the esophageal lesions revealed that an increase in the number of infused MDSCs in the tumor were associated with an increased incidence of esophageal invasive carcinoma formation (Fig. 3b-c). These data suggest that the increased number of MDSCs promoted tumor progression in carcinoma.

Induction of angiogenesis and suppression of T cell proliferation was reported to play a key role in tumor promotion by MDSCs [21, 24]. As shown in Figure 3d, the MDSC infusion attenuated CD8+T cell accumulation and enhanced CD31 and VEGF immunostaining.

### Levels of circulating IL-6 correlate with tumor progression and the percentage of CD11b^+^CD14^+^HLA-DR^−^ cells in cancer patients

**Figure 4:**
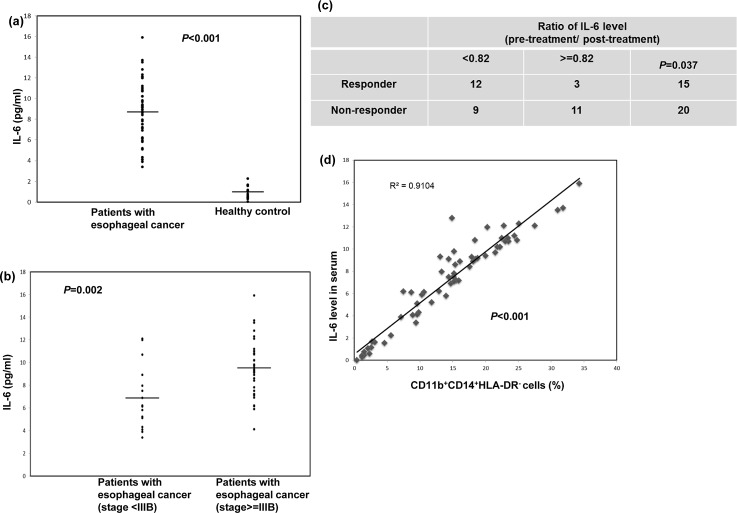
Circulating IL-6 levels correlated with tumor progression and CD11b^+^CD14^+^HLA-DR^−^ cells accumulation in cancer patients a. IL-6 levels were measured using ELISA in plasma samples obtained from the cancer patients (n=50) and the healthy donors (n=12). The lines indicate the mean values. b. IL-6 levels measured using ELISA in plasma samples obtained from cancer patients with stage II and IIIA (n=15) and stage IIIB and IV (n=35) disease. The lines indicate the mean values. c. Differences in treatment response according to the mean IL-6 levels after treatment relative to those at diagnosis (82%) in the high (ratio after treatment ≥ 82%) and low groups (< 82%).(The ratio means post-treatment IL-6 level / IL-6 level at diagnosis). d. CD11b^+^CD14^+^HLA-DR^−^ myeloid cells were obtained, as previously described, from 50 cancer patients and 12 healthy controls; the plasma IL-6 levels were then compared between the groups.

IL-6 is associated with tumor progression *in vivo* and in clinical patients for certain cancers [20, 25, 26]. To further investigate the potential correlation of IL-6 levels with esophageal tumor progression, we examined the levels of IL-6 in the PB samples from the 50 SCC patients and 12 healthy controls using ELISA. The mean IL-6 level in esophageal SCC patients was 8.73±2.85 ng/ml, compared with 0.99±0.67 ng/ml in the healthy donors (Fig. 4a). In addition, the IL-6 levels were higher in patients with stage IIIb or IV disease compared with stage II or IIIa disease (Fig. 4b). To analyze this further, the 35 patients who completed the planned treatment were divided into two groups according to their mean IL-6 level after treatment relative to the level at diagnosis (82%): high (ratio after treatment ≥ 82%) and low groups (< 82%). As shown in Figure 4c, 12 of the 21 patients in the low group were responders, but only three patients in the high group showed a response to treatment. Moreover, there was a strong correlation between the IL-6 levels and the percentage of CD11b^+^CD14^+^HLA-DR^−^ cells in the clinical patients (Fig.4d).

### Effects of circulating IL-6 on tumor growth and MDSC accumulation *in vivo*

**Figure 5:**
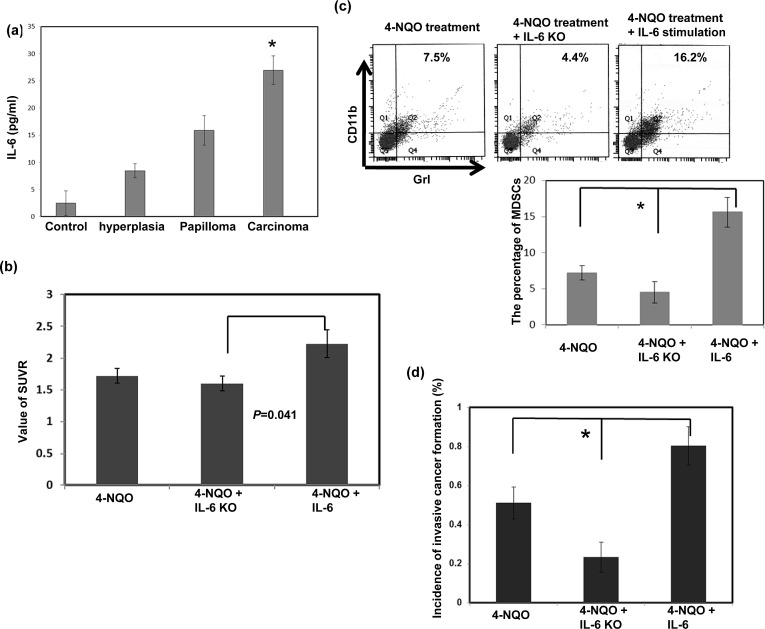
Effect of circulating IL-6 levels on tumor progression and MDSC accumulation *in vivo* a. IL-6 levels in the mice diagnosed with hyperplasia/papilloma or invasive carcinoma were measured using ELISA. Quantitative data are shown. The columns represent the means ± SEM. *, p<0.05. b. Loss of IL-6 decreased the SUVR in the PET images of esophageal lesions in IL-6 KO mice compared with that in the 4-NQO- treated C57 mice with IL-6 stimulation. The tumor-to-muscle SUVR was calculated from the micro-PET scans. Each point represents the means ± SEM. *, p<0.05 c. Flow cytometry analysis of CD11b+Gr1+ cells in the C57 or IL-6 KO mice after treatment with 4-NQO. Representative images and quantitative data are shown. Each column represents the mean ± SD. *, p<0.05 d. Quantitation of the incidence of esophageal tumors with invasive carcinoma using the pathological examination of sectioned tissue samples of the C57 or IL-6 KO mice after treatment. Each column represents the means ± SEM. *, p<0.05.

We used ELISAs to measure the circulating levels of IL-6 in the 4-NQO-induced esophageal tumor mouse model. As shown in Figure 5a, the mice that had esophageal tumors with invasive carcinoma had significantly higher serum IL-6 levels than those with hyperplasia or papilloma alone. The role of IL-6 in MDSC accumulation and tumor progression was further examined using the 4-NQO-induced esophageal tumor model in the presence or absence of IL-6. Figure 5b showed that the absence of IL-6 decreased the SUVR in PET images of the esophageal lesions of the IL-6 KO mice compared to that of the C57 mice with IL-6 stimulation. Histological examination and FACS analysis of the esophageal lesions revealed that an increase in IL-6 augmented the recruitment of MDSCs and the formation of esophageal invasive carcinoma (Fig. 5c-d). These data suggest that IL-6 was associated with MDSC accumulation and invasive tumor progression.

### Effects of IL-6 on the induction of MDSCs

**Figure 6:**
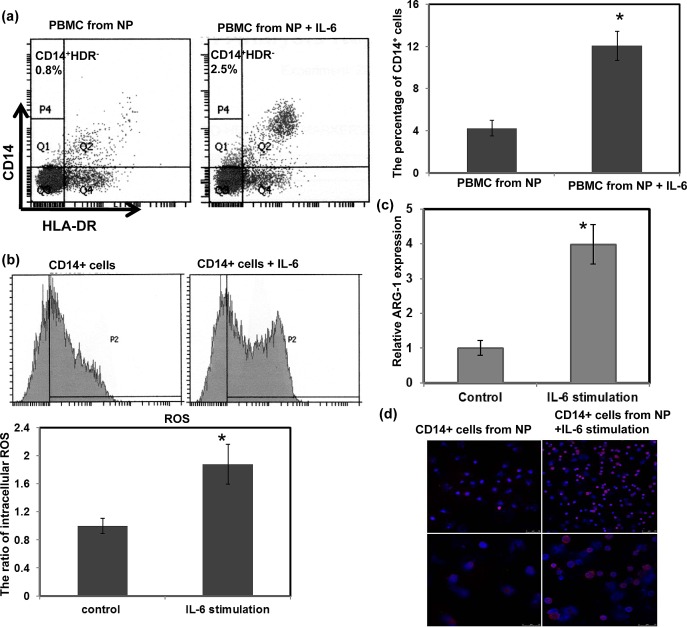
Effect of IL-6 on the induction of MDSCs (a) Flow cytometric analysis of CD14^+^HLA-DR^−^ cells among PBMCs from healthy controls treated with or without 10 ng/ml IL-6 for 1 week. IL-6 stimulation increased the percentage of CD14+HLA-DR− MDSCs. (b) CD14+ cells isolated from IL-6-stimulated PBMCs showed higher intracellular ROS levels. (c) Increased ARG1 expression was measured using real-time RT-PCR. Each column represents the means ± SEM. *, p<0.05. (d) Immunofluorescent staining revealed greater levels of activated STAT3 in IL-6-treated cells than that in the untreated cells. (DAPI, blue; p-STAT3, red) (Upper row, low power field; lower row, high power field)

The data described above suggest that the levels of IL-6 significantly correlated with the number of MDSCs in the esophageal cancer patients and SCC animal models. Therefore, we directly assessed the role of IL-6 signaling in MDSC induction using PBMCs from healthy controls. The PBMCs were cultured in complete medium in the presence or absence of 10 ng/ml recombinant IL-6 for 7 days. FACS analysis demonstrated a high frequency of CD14^+^HLA-DR^−^ myeloid cells induction among the IL6-treated PBMCs (Fig. 6a). MDSCs have been reported to inhibit T cell effector function via a range of mechanisms including reactive oxygen species (ROS) production and increased arginase 1 (ARG1) levels [24, 27, 28]. Therefore, to further confirm IL-6 induction of functional MDSCs, the levels of ARG1 and ROS were examined. In addition, STAT3 activation was reported to regulate ARG1 in MDSCs isolated from cancer patients. As shown in Figure 6b–d, increased levels of ARG1, ROS, and activated STAT3 were detected in IL-6-treated CD14^+^ cells. These data suggest that IL-6 plays a critical role in the induction of MDSCs.

## DISCUSSION

Tumor-induced immune suppression in cancer patients is a major issue that not only promotes tumor progression but also inhibits the efficiency of cancer treatment [14, 24]. MDSC are thought to promote cancer progression and present an important barrier limiting the full potential of immune-based cancer therapies or the endogenous host response against tumor development [29]. Additionally, they have been associated with the prognoses of some cancers [4, 5, 25, 30]. Although GI malignancies are strongly associated with inflammation, and increased numbers of MDSCs have been associated with an increased risk of death [11, 31, 32], the correlation between esophageal SCC and MDSCs requires further investigation.

To date, MDSCs have been defined mainly as HLA-DR−, CD11b+, CD33+, and CD15+ lineages in human cancers [33, 34]. A novel, well-characterized subset of human MDSCs is monocytic CD14+ cells, which express decreased levels of HLA-DR. The CD14^+^HLA-DR^-^ subset of MDSCs was reported to contribute to tumor progression and treatment resistance in non-small lung cancer and head and neck SCC [13, 14, 35]. In the present study, we characterized the percentage of CD11b^+^CD14^+^HLA-DR^-^ myeloid cells in a cohort of esophageal SCC patients. FACS analysis revealed that the number of CD11b^+^CD14^+^HLA-DR^-^ cells significantly increased in the PB of the esophageal SCC patients compared with the healthy donors. Moreover, the proportion of CD11b^+^CD14^+^HLA-DR^-^ cells correlated with disease status and could predict the treatment response.

To further investigate the link between the recruitment of MDSCs and tumor progression, we induced esophageal tumors in immunocompetent mice using 4-NQO [16]. The model mimics many features observed human esophageal SCC development; therefore, this model should be advantageous for studies of esophageal tumor development. The co-expression of the myeloid cell lineage differentiation antigens GrI and CD11b was assessed to characterize MDSCs in the mice [6, 21]. Over the 12–14 week period following the 16-week 4-NQO treatment, esophageal lesions were visualized using micro-PET and gross examination. The examination of tissue sections from the 4-NQO-treated mice revealed the presence of lesions in the esophagus, including hyperplasia, papilloma, and invasive carcinoma. The SUVR, detected using micro-PET, was significantly higher in those esophageal lesions with invasive carcinoma compared with those with only hyperplasia and/or papilloma. Moreover, the percentage of MDSCs was correlated with tumor progression in mice, consistent with clinical data. There was significantly higher MDSC recruitment in mice with esophageal SCC compared with control mice. To provide direct evidence for the relationship between MDSC recruitment and tumor progression, we assessed tumor progression in 4-NQO-treated mice infused with sorted CD11b^+^GrI^+^ cells. The expansion of MDSCs induced by intravenous infusion was associated with a higher SUVR in esophageal lesions and an increased incidence of invasive esophageal tumor formation.

MDSCs are potent suppressors of T cell proliferation and activation. The ability of MDSCs to suppress T cell proliferation may be central to tumor formation [4, 5, 24]. In addition to immunosuppression, bone marrow-derived CD11b^+^ myeloid cells mediate several myeloid cell functions, including migration and angiogenesis [36, 37]. The recruitment of various blood-borne bone marrow-derived cells might be important for tumor neovascularization. Immunofluorescent staining in the current study revealed decreased CD8+ T cell infiltration, and increased angiogenesis (CD31 and VEGF staining) was observed in the esophageal lesions of the MDSC-infused mice. These findings suggest that MDSC recruitment could promote esophageal cancer formation via immune suppression and angiogenesis.

The accumulation and activation of MDSCs is driven by multiple factors, several of which are associated with chronic inflammation, notable IL-6, IL-1β, IDO and activated STAT3 [4, 5, 8, 18, 19, 35]. Activation of STAT3 has been shown to enhanced the expression of IDO, a key factor maintaining immune tolerance in myeloid cells, and is the main transcription factor that regulates the expansion of MDSC [38]. Importantly, STAT3 is prominently activated at sites of chronic inflammation by IL-6 and initiates a positive that is highly predisposing condition for cancer [39]. IL-6 is a multifunctional cytokine which plays an important role in a wide range of biologic activities in different types of cell including inflammatory cells and tumor cells. As shown in previous studies, IL-6 and IL-1β are associated with the prognosis of esophageal SCC [20, 40, 41]. Moreover, MDSCs do express IL-6R [19, 42]. Tumor-secreted IL-6 has been reported to restores MDSC accumulation and is a downstream mediator of the IL-1β–induced expansion of MDSC. Therefore, we hypothesized that IL-6 plays a critical role in the induction of MDSCs in patients with esophageal SCC. We assessed the relationship between plasma IL-6 levels and tumor progression by assessing MDSC levels in clinical patients with esophageal SCC and in 4-NQO-treated mice. IL-6 levels were elevated significantly in cancer patients compared with healthy donors. Moreover, there was a significant correlation between plasma IL-6 levels and the percentage of CD11b^+^CD14^+^HLA-DR^-^ myeloid cells.

To further examine the ability of IL-6 to induce MDSCs, mononuclear cells from the PB of healthy donors were cultured in medium in the presence or absence of IL-6 stimulation. FACS revealed that IL-6 stimulation increased the percentage of CD14^+^HLA-DR^-^ myeloid cells. It was reported that ARG-1-mediated depletion of L-arginine, ROS production, and the overexpression of VEGF regulate the functions of MDSCs [20, 23]. Furthermore, STAT3 activation regulates the suppressive function of ARG-1 in MDSCs [35]. We demonstrated that the IL-6-stimulated CD14+ cells expressed significantly higher levels of ARG-1, ROS, and p-STAT3 compared with control CD14+ cells sorted from the PBMCs of the healthy donors. The data revealed that IL-6 plays an important role in the induction of MDSCs.

Novel immune-based therapies for the treatment of cancer are currently under development. Immunotherapy using recombinant antibodies and vaccines is emerging as a promising treatment approach for several solid tumors, as well as a number of GI malignancies. Many of these approaches involve immunotherapy and are likely to be most effective in immunocompetent tumor-bearing individuals who are minimally immunosuppressed. Therefore, we examined the effect of IL-6 on tumor progression in the 4-NQO-treated mice. The C57 mice with IL-6 stimulation showed significantly increased MDSC levels, which was associated with a higher SUVR in the esophageal lesions and an increased incidence of invasive esophageal tumor formation. Conversely, blocking IL-6 abrogated the induction of MDSCs and the incidence of 4-NQO-induced invasive tumors. These findings suggest that the IL-6-mediated induction of MDSCs was associated with esophageal tumor promotion and poor prognosis. In the present study, the animal model provides the direct evidence on the link between IL-6, MDSCs accumulation and esophageal tumor formation. However, different MDSC subsets may play a different role in this environment. In the induced esophageal tumor animal model, the protracted time of onset, the variability in timing and incidence of tumor development is a significant limitation in providing robust evaluation. Future studies are needed in shaping the MDSC composition and function.

In summary, the levels of MDSC and IL-6 predicted the prognosis and treatment response of patients with esophageal SCC. Moreover, IL-6-induced MDSC recruitment provides a microenvironment conducive to tumor growth and the development of treatment resistance. Therefore, targeting IL-6 signaling such as CK2 inhibitor and rapamycin [43, 44] could be a promising strategy for the treatment of esophageal cancer. The issue should be best answered in context of a prospective study in a more patient population.

## MATERIALS AND METHODS

### Patient characteristics

The Institutional Review Board of our hospital approved this study. Peripheral blood (PB) samples were obtained from 50 patients with pathologically and clinically confirmed esophageal SCC. All patients with malignancy received complete staging. None of the subjects with esophageal SCC had been treated with chemotherapy, radiotherapy, or surgery before sampling. In addition, 12 healthy donors were recruited as controls. Whole blood samples were obtained from all subjects and were analyzed within 6 h of collection. The planned treatments for the esophageal cancer patients included concurrent chemoradiotherapy (CCRT) or pre-operative CCRT combined with surgery according to the guidelines proposed by the oncology team at our hospital. After the completion of treatment, the patients underwent a repeat computed tomography (CT) scan and endoscopic examination to determine the treatment response. In the present study, the responders were defined as the patients with a complete pathological response or a partial response with ≥ 50% reduction in tumor size. Of the 50 cancer patients, 35 completed the treatment course and donated a second blood sample for MDSC analysis 2 months after completing treatment to avoid treatment-induced effects.

### MDSC isolation and flow cytometry analysis

To assess the frequency of MDSCs among peripheral blood mononuclear cells (PBMC) in the patients, multicolor fluorescence-activated cell sorting (FACS) was performed using a FACS caliber flow cytometer (BD Biosciences). The human MDSC subset characterized as CD11b^+^CD14^+^ HLA-DR^−^ was sorted from freshly obtained PB. The leukocytes were separated from the PB using a Ficoll gradient prior to analysis or sorting. Multicolor cell analysis was performed using the following antibodies: PerCP-Cy™5.5-conjugated CD14, PE-conjugated CD11b, and FITC-conjugated HLA-DR. The co-expression of the myeloid-cell lineage differentiation antigens GrI and CD11b has been previously characterized in MDSCs from mice [21]. FACS was performed on single-cell suspensions prepared from whole tumors and the spleens of the mice after digestion and immunostaining for CD11b and GrI using fluorescence-labelled monoclonal antibodies (BD Pharmingen). The percentage of MDSCs was measured using multicolor flow cytometry, and isotype-specific antibodies were used as negative controls.

### Induction of esophageal tumors in mice

Six-week-old male C57BL/6 and IL-6 knockout mice on a C57BL/6J background (B6.129S2-Il6tm1Kopf/J) (IL-6 KO) were used for the 4-NQO-induced esophageal tumor model [16]. A stock solution of the carcinogenic 4-NQO (Sigma, St. Louis, MO, USA) was prepared weekly in propylene glycol and added to the drinking water of the mice at a concentration of 100 μg/ml. The mice were allowed access to the drinking water containing 4-NQO (tumor-induced group) or the solvent only (control group) at all times during the treatment. After 16 weeks of treatment, the mice were analyzed for lesions in the esophagus at different times up to 14 weeks. The Experimental Animal Ethics Committee of our hospital approved this protocol. The body weights were measured, and blood was drawn for IL-6 quantification at the indicated times. To analyze the effects of MDSC infusion, sorted MDSC cells (1 × 10^6^ CD11b^+^Gr I^+^ cells per mouse) were injected intravenously (once per week, eight times) 12 weeks after the initiation of the 4-NQO treatment. To further assess the effect of IL-6 blocking, the IL-6 KO mice were used with the 4-NQO-induced esophageal tumor model to analyze tumor growth in a microenvironment lacking IL-6. For IL-6 stimulation *in vivo*, an i.p injection of IL-6 (100 ng per mice, thrice a week for 6 weeks) was started 12 weeks after the initiation of the 4-NQO treatments.

### Small-animal PET Imaging of tumor

4-NQO-induced esophageal tumors were detected using PET [22] following the injection of 7.4–9.25 MBq of 2-deoxy-2-[F-18]fluoro-D-glucose (FDG) via the tail vein 60 minutes prior to the PET scan, which was performed on an Inveon™ system (Siemens Medical Solutions Inc., Malvern, PA, USA) located in the Molecular Image Center of our hospital. All mice underwent micro CT imaging for anatomic registration following the micro-PET imaging. The esophageal tumor regions of interest (ROIs) and the non-lesion ROIs from the muscle adjacent to the esophagus were selected as the areas with 20% maximum activity in the sagittal plane using PMOD version 3.2 image analysis software (PMOD Technologies Ltd., Zurich, Switzerland). The mean radioactivity concentration within the tumor or organ was obtained from the mean pixel values within the multiple ROI volume and was expressed as the standardized uptake value. The standard tracer uptake value ratio (SUVR, tumor-to-muscle) was calculated to represent the tumor lesion for statistical comparisons.

After the PET examination, gross and tissue examinations were performed to evaluate the esophageal lesions in the 4-NQO-treated mice. The gross lesions were identified and photographed. A pathologist analyzed the esophageal lesions histologically. The examination of the tissue sections revealed pathologies including hyperplasia (thickened epithelium with prominent surface keratinization), papilloma (noninvasive exophytic growth of neoplastic cells), and invasive carcinoma (lesion with invasion into the subepithelial tissues).

### Immunofluorescence staining (IF)

Frozen tissue specimens were cut into 5 × 8-μm cryostat sections and then brought to room temperature for IF staining. MDSCs were seeded onto cover slips and either treated or left untreated. Next, they were fixed at the indicated times, permeabilized with 2% paraformaldehyde for 5 min, and washed with PBST. The slides were incubated for 1 h at room temperature with antibodies against p-STAT3, VEGF, and CD31, followed by 1 h with a FITC-conjugated secondary antibody. The slides were counterstained with DAPI to visualize the nuclei. After two washes with PBST, specific target proteins were visualized using a fluorescence microscope.

### Enzyme-linked immunosorbent assay analysis of IL-6 levels

IL-6 levels in human and murine serum samples were analyzed using an IL-6 human and mouse Quantikine ELISA kit (R&D system). To measure circulating IL-6 levels, blood was removed from the peripheral circulation of cancer patients and from the heart of mice. The samples were frozen and stored before assaying.

### Induction of CD14^+^ HLA-DR^−^myeloid cells from PBMC

The ability of IL-6 to induce the generation CD14^+^ HLA-DR^−^myeloid cells was evaluated from healthy donor PBMC. Briefly, PBMCs were cultured for 1 week in the presence or absence of 10ng/ml IL-6, then the proportion of CD14^+^HLA-DR^-^ myeloid cells were analyzed by FACS, and CD14+ cells were isolated for the following tests.

### Real-time reverse transcription-polymerase chain reaction (RT-PCR)

Real-time RT-PCR was performed on RNA extracted from cell cultures. Quantative RT-PCR for ARG-1 was performed using SYBR green qPCR mastermix. The primer sequences were as follows: (forward and reverse, respectively) 5′-GTTTCTCAAGCAGACCAGCC -3′ and 5′-GCTCAAGTGCAGCAAAGAGA-3′ for ARG-1 [23]. A β-actin primer set was used as a loading control. The optimized PCR was performed on an iCycler iQ Multicolor Real-Time PCR detection system. Significant fluorescent PCR signals from carcinoma tissue were normalized relative to the mean value of signals obtained from control samples.

### Intracellular reactive oxygen species (ROS) generation

Intracellular ROS was assayed using a fluorescent dye, 2′7′-dichlorofluorescein diacetate (DCFH-DA). Briefly, 5×10^5^ CD14^+^ cells were incubated in phenol-red free and serum-free medium containing 20μM DCFH-DA for 1 hour to measure ROS production. In the presence of ROS, DCFH is oxidized to the highly fluorescent DCF. DCF fluorescence was measured using a flow cytometer equipped with a 488 nm argon laser.

### Statistical analysis

The significance of differences between samples was determined using Student's t-tests. Data are presented as the means ± standard error of the mean (SEM). All experiments, comprising three replicates, were performed at least twice independently. A probability level of p < 0.05 was adopted throughout to determine statistical significance, unless otherwise stated.

### Competing interests

The authors confirm that there are no conflicts of interest that could be perceived as prejudicing the impartiality of the research reported
